# Phage P2-71 against multi-drug resistant *Proteus mirabilis*: isolation, characterization, and non-antibiotic antimicrobial potential

**DOI:** 10.3389/fcimb.2024.1347173

**Published:** 2024-03-04

**Authors:** Zhiyou Dong, Ruihu Wu, Lijuan Liu, Shengquan Ai, Jinpeng Yang, Qianlan Li, Keyi Fu, Yunian Zhou, Hualin Fu, Ziyao Zhou, Haifeng Liu, Zhijun Zhong, Xianmeng Qiu, Guangneng Peng

**Affiliations:** ^1^ Key Laboratory of Animal Disease and Human Health of Sichuan Province, College of Veterinary Medicine, Sichuan Agricultural University, Chengdu, Sichuan, China; ^2^ New Ruipeng Pet Healthcare Group, Chengdu, China

**Keywords:** bacteriophage therapy, multidrug-resistant proteus mirabilis, non-antibiotic antimicrobial strategies, phage genomics and host interactions, phage-bacteria interaction

## Abstract

*Proteus mirabilis*, a prevalent urinary tract pathogen and formidable biofilm producer, especially in Catheter-Associated Urinary Tract Infection, has seen a worrying rise in multidrug-resistant (MDR) strains. This upsurge calls for innovative approaches in infection control, beyond traditional antibiotics. Our research introduces bacteriophage (phage) therapy as a novel non-antibiotic strategy to combat these drug-resistant infections. We isolated P2-71, a lytic phage derived from canine feces, demonstrating potent activity against MDR *P. mirabilis* strains. P2-71 showcases a notably brief 10-minute latent period and a significant burst size of 228 particles per infected bacterium, ensuring rapid bacterial clearance. The phage maintains stability over a broad temperature range of 30-50°C and within a pH spectrum of 4-11, highlighting its resilience in various environmental conditions. Our host range assessment solidifies its potential against diverse MDR *P. mirabilis* strains. Through killing curve analysis, P2-71’s effectiveness was validated at various MOI levels against *P. mirabilis* 37, highlighting its versatility. We extended our research to examine P2-71’s stability and bactericidal kinetics in artificial urine, affirming its potential for clinical application. A detailed genomic analysis reveals P2-71’s complex genetic makeup, including genes essential for morphogenesis, lysis, and DNA modification, which are crucial for its therapeutic action. This study not only furthers the understanding of phage therapy as a promising non-antibiotic antimicrobial but also underscores its critical role in combating emerging MDR infections in both veterinary and public health contexts.

## 1 Introduction

*P. mirabilis*, a Gram-negative bacterium, is renowned for its remarkable swarming motility on solid surfaces. This bacterium is a frequent urinary tract pathogen, particularly among patients with prolonged catheter usage (O’Hara et al., 2000; Schaffer and Pearson, 2015). Besides urinary tract infections (UTIs), *P. mirabilis* also causes other infections including superficial skin and respiratory tract infections (Jacobsen and Shirtliff, 2011). The escalating emergence of MDR strains of *P. mirabilis* underscores the pressing need to discover new and effective approaches for combating microbial infections (Algammal et al., 2021; Kang et al., 2021; Li et al., 2022). The emergence of MDR *P. mirabilis* strains in dogs presents a significant public health concern, particularly due to the close relationship between dogs and humans (Decôme et al., 2020; El-Tarabili et al., 2022).

The presence of MDR bacteria in UTIs reduces the efficacy of antibiotic treatments (Flores-Mireles et al., 2015). Phages have emerged as potentially promising alternatives for managing antibiotic-resistant UTIs. Recent research has shown that phages can effectively target bacteria in UTIs and disrupt their biofilms (Melo et al., 2016; Yazdi et al., 2018). Scientists suggest using phage therapy as a strategy to prevent and treat UTIs caused by MDR bacteria. However, phage-resistant strains continue to emerge despite the use of phage cocktails and phage-antibiotic combinations (Chegini et al., 2021; Oyejobi et al., 2022). To effectively address this problem, further foundational studies are essential to explore the interplay between phages and their hosts and the potential of phages in managing UTIs. Additionally, understanding the stability and activity of phages within the challenging environment of the urinary tract, especially in artificial urine which simulates the conditions of the urinary system, is vital for the development of effective phage therapy.

In this study, we isolated a lytic *P. mirabilis* phage named P2-71 from dog feces. We conducted a comprehensive evaluation that included transmission electron microscopy for morphology assessment, host range determination, one-step growth curve analysis, temperature and pH stability assessments. Additionally, we performed killing curve analyses in both LB broth and artificial urine to characterize its bactericidal properties. A detailed genomic analysis of P2-71 was also undertaken. Our extensive research into P2-71 highlights its potential as an effective non-antibiotic antimicrobial agent, demonstrating its relevance in addressing the pressing global issue of drug-resistant bacterial infections.

## 2 Materials and methods

### 2.1 Experimental bacterial strains

This investigation employed MDR *P. mirabilis* strains retrieved from dog fecal matter, which are maintained within our lab’s repository. *P. mirabilis* 37, serving as the host strain for phage P2-71, was among these strains. The multi-drug resistance (MDR) status of these *P. mirabilis* strains was determined based on resistance to at least three different classes of antimicrobial agents, as per the standards set by the Clinical and Laboratory Standards Institute (CLSI). Details regarding the bacterial strains, their origins, the accession numbers correlating to the 16S rRNA gene sequences, and the specific resistance profiles can be found in (Liu et al., 2023). A brief summary of the strains used is provided in (**Supplementary Table 1**). Additionally, four standard strains - *Salmonella enterica* H9812, *Pseudomonas aeruginosa* PAO1, *Staphylococcus aureus* ATCC, 25923, and enterotoxigenic *Escherichia coli* (ETEC) ATCC, 25922 - were procured from the American Type Culture Collection (ATCC).

To initiate bacterial growth, we cultured the strains in Lysogeny Broth (LB) and then maintained them in an incubator at a stable 37°C with agitation at 160 rpm throughout the night to promote favorable growth conditions.

### 2.2 Isolation and purification of phage P2-71

The phage P2-71, retrieved from dog fecal matter from the Protect Beastie Facility in Sichuan, underwent an extraction process starting with the blending of 1 gram of fecal content with 1 milliliter of SM buffer. This blend was then subjected to centrifugation at, 8000×g for a duration of 5 minutes at a temperature of 4°C, and the supernatant obtained was passed through a filter with 0.22 μm pores to discard solid particles. This clarified supernatant was then combined with a logarithmic-phase *P. mirabilis* 37 culture and maintained for 6 hours at a temperature of 37°C with continuous shaking at 220 revolutions per minute. Post-incubation, a centrifuge run at, 12000×g for 3 minutes at 4°C was performed to separate the supernatant. This supernatant was then diluted in a gradient, mixed equally with a bacterial culture in the logarithmic-phase, and set aside for a quarter of an hour. Afterward, the diluted mixture was incorporated into 4 milliliters of a semi-solid LB substrate employing the double agar layer technique, before being applied to firm LB agar bases. These bases were then kept in an incubator set at a stable 37°C for an overnight period. Distinct plaques, which signify phage activity, were meticulously extracted into SM buffer and amalgamated thoroughly. To guarantee a pure phage specimen, this meticulous extraction was conducted multiple times.

The purification and storage of phage P2-71 were modified based on the method described by Mondal et al (Mondal et al., 2023), starting with the mixture of the *P. mirabilis* 37 logarithmic-phase culture with phage P2-71 at an MOI of 0.1, followed by the addition of 2 ml of LB medium. This mixture was then incubated overnight at 37°C with constant shaking at 220 rpm. After this period, a centrifugation at, 8000×g for 10 minutes at 4°C was performed, and the supernatant was kept. The sample was then subjected to an ultracentrifugation at, 30000 rpm for 4 hours at 4°C. Subsequently, the pellet was resuspended and subjected to sucrose density gradient centrifugation under identical conditions for 4 hours, which facilitated the collection of the phage band. This collected phage layer was then diluted twofold and underwent a low-speed centrifugation at, 8000×g for 10 minutes at 4°C to extract excess sucrose. This step of sucrose removal was repeated three time. The final concentrated phage P2-71 was stored at a refrigeration temperature of 4°C.

### 2.3 Host range

To assess the host range of phage P2-71, logarithmic-phase bacterial cultures and a freshly prepared phage solution were used. Serial dilutions (gradient dilutions) of the phage were prepared, and these were mixed with the bacterial cultures in a 1:1 ratio, allowing the mixtures to stand for 15 minutes to facilitate interaction. Subsequently, each mixture was combined with 4 mL of semi-solid LB medium and carefully overlaid onto solid LB medium plates. These plates were then placed in a constant-temperature incubator at 37°C and incubated overnight. The presence of clear zones or plaques on the plates indicated bacterial susceptibility to lysis by phage P2-71. Bacterial strains that displayed such clear plaques were classified as susceptible to the phage, evidencing the lytic capability of phage P2-71 against these bacterial hosts.

### 2.4 Biological characteristics of phage P2-71

Investigation into the structural composition of phage P2-71 was carried out through a transmission electron microscope (TEM) 2100plus (JEOL, Japan), adhering to sample preparation guidelines outlined in established research (Hao et al., 2023).

A one-step growth curve was utilized to determine the latent period, rise phase, plateau period, and burst size of phage P2-71. Newly reactivated phage particles at a multiplicity of infection (MOI) of 0.01 were amalgamated with an actively dividing *P. mirabilis* 37 culture (2x10^8^ CFU/mL), followed by a resting period of 15 minutes to facilitate phage-bacteria engagement. Post-rest, the blend was centrifuged at 13,000×g for a brief span of 2 minutes to segregate non-adsorbed phages. The supernatant was then removed, and the resultant pellet was reintroduced into LB broth and incubated at 37°C with a shaker set at 220 rpm, marking the commencement of the observation as zero time. Sampling was done initially at 5-minute intervals for the first 20 minutes, followed by 10-minute intervals, with immediate gradient dilution for phage titer analysis. The burst size was determined by the quotient of the released phage count to the initial infected bacterial cell count during the latency phase (D’Andrea et al., 2017).

The pH stability of phage P2-71 was ascertained through a battery of pH conditions ranging from 1.0 to 14.0 in SM buffer. Each buffer, containing the phage at a starting titer of 10^9^ PFU/mL, was incubated at a stable 37°C for an hour in a temperature-controlled water bath. Subsequent titer evaluations were performed employing the double layer agar plate technique.

The thermal endurance of the phage was gauged by exposing phage suspensions at a concentration of 10^9^ PFU/mL to varied constant heat settings (30, 40, 50, 60, 70, and 80°C) for periods ranging from 30 to 60 minutes. Determination of phage concentrations post-heat exposure was standardized to the 30°C, 60-minute control. To ensure the validity of the results, each assessment was replicated threefold.

### 2.5 Time-kill assay

The *in vitro* bactericidal activity of phage P2-71 was evaluated using a time-kill assay, adapted from the method described by (Ciacci et al., 2018, 101). The phage titer was adjusted to create a series of MOI levels including 10, 1, 0.1, and 0.01. For each MOI, 100 μl of phage solution was mixed with 100 μl of *P. mirabilis* 37 in the logarithmic growth phase (2 × 10^8^ CFU/mL). The resulting mixtures were then transferred into 10 ml of LB liquid culture medium. These cultures were incubated at 37°C with constant agitation at 220 rpm. The optical density at 600 nm (OD600) was measured at 10-minute intervals using a Thermo Varioskan Flash spectral scanning multimode reader (Thermo Fisher Scientific, United States). This procedure was performed in triplicate to ensure the reproducibility of the results.

### 2.6 Stability of phage P2-71 in artificial urine

The stability of phage P2-71 was evaluated in an artificial urine solution (Phygene, Fuzhou, China) with a pH of 5.7, to closely replicate the urinary tract environment. After the inoculation of the phage, the suspension was maintained at a steady temperature of 37°C for incubation. To assess the stability of P2-71, samples were collected at specified time intervals: 0, 1, 2, 4, 6, 12, 18, and 24 hours post-inoculation. At each time point, the titer of phage P2-71 was quantified using the double-layer agar plate method. This assay was designed to understand the persistence and stability of P2-71 in a simulated urinary environment, providing insights into its potential for clinical applications.

### 2.7 Time-kill assay of P2-71 in artificial urine

Following the protocol outlined in Section 2.5, the bactericidal effect of phage P2-71 on *P. mirabilis* 37 was further examined in an artificial urine environment, aiming to simulate conditions similar to the urinary tract. Cultures of *P. mirabilis* 37 in their logarithmic growth phase were first centrifuged at, 5000×g for 20 minutes at 4°C. After discarding the supernatant, the bacterial pellet was resuspended in PBS. This centrifugation and resuspension process was repeated once more to ensure thorough removal of the LB medium. The bacterial concentration was then adjusted to 2 × 10^8^ CFU/mL, readying it for combination with phage P2-71 at MOI levels of 10, 1, 0.1, and 0.01.

These mixtures were subsequently transferred into 10 ml of artificial urine and incubated in a temperature-controlled shaker at 37°C, with continuous agitation at 220 rpm. The interaction between the phage and bacteria was monitored for a period of 180 minutes, and OD600 was measured at 10-minute intervals. Each set of conditions was replicated in triplicate to ensure the consistency and reproducibility of the experimental data.

### 2.8 Genomic analysis

The genomic sequencing of phage P2-71 was executed utilizing the PE150 module on the NovaSeq, 6000 apparatus. Subsequent bioinformatics analysis was undertaken, initiating with Fastp (Chen et al., 2018) for the primary data processing which encompassed adapter trimming, discarding low-quality sequences, and filtering out high N ratio reads. The *de novo* assembly was conducted via metaSPAdes (Nurk et al., 2017), with a series of k-mer lengths tested to achieve optimal results. Alignment of clean reads to the assembled genome for coverage assessment was done using bwa (Li and Durbin, 2009).

Annotation of open reading frames (ORFs) was conducted using Rapid Annotation using Subsystem Technology (RAST) (Brettin et al., 2015) and Prokka (Seemann, 2014), complemented with blastp for **Supplementary Annotations**. The Virulence Factor Database (VFDB) (Liu et al., 2022) was utilized for virulence factors identification, while predictions for potential drug-resistance genes were made through the Comprehensive Antibiotic Resistance Database (CARD) (Alcock et al., 2023). tRNA genes were forecasted using tRNAscan-SE 2.0 (Chan et al., 2021), and CRISPR arrays along with Cas proteins were identified via CRISPRCasFinder (Couvin et al., 2018). Visualization of the phage P2-71 genomic map was facilitated by Proksee (Grant et al., 2023).

Phylogenetic investigations were conducted with MEGA11 software (Tamura et al., 2021, 11), particularly focusing on the terminase large subunit gene of phage P2-71 and its closely related sequences. The Neighbor-Joining method (Saitou and Nei, 1987), was employed to construct the phylogenetic tree, and the tree’s robustness was verified through, 1000 bootstrap tests (Felsenstein, 1985). The calculation of evolutionary distances was executed using the Maximum Composite Likelihood approach (Tamura et al., 2004). This analysis included 16 sequences, spanning, 2012 positions after removing all ambiguous bases, with pairwise deletion as the chosen strategy. Comparative genomic insights were graphically portrayed using Easyfig 2.2.5 (Sullivan et al., 2011). Accession numbers for the phage genomes included in this phylogenetic analysis are available in (**Supplementary Table 2**).

## 3 Result

### 3.1 The characteristics of phage P2-71

**Figure 1:**
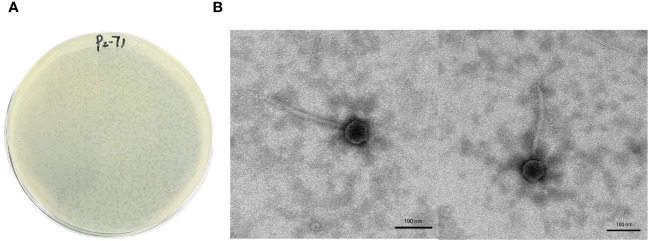
Morphological analysis of phage P2-71. **(A)** Plaque Morphology of Phage P2-71: This panel shows a Petri dish following the Double-Layer Agar technique used for phage isolation, displaying the characteristic small, circular, and translucent plaques formed by phage P2-71 against a lawn of *P. mirabilis* 37. These plaques are indicative of lytic activity where the phage has infected and cleared areas of bacterial growth on LB agar medium. **(B)** Presented here are high-resolution TEM images of phage P2-71, which unveil its structural morphology. The phage is observed to have a dodecahedral head, approximately 62 ± 3.0 nm in diameter, attached to a lengthy tail measuring about 234 ± 10 nm. These dimensions and shape are typical features of phages belonging to the Siphoviridae family, suggesting its classification within this group. Scale bars represent 100 nm, providing a reference for size estimation.

In the current investigation, phage P2-71 was successfully isolated from *P. mirabilis* 37. The isolation technique employed the Double-Layer Agar method, resulting in the formation of distinct, small circular, and translucent plaques (**Figure 1A**). Transmission electron microscopy (TEM) provided detailed insights into the morphology of phage P2-71, illustrating a dodecahedral head with an average diameter of 62 ± 3.0 nm and a tail measuring an average length of 234 ± 10 nm (**Figure 1B**), characteristic of the Siphoviridae family.

### 3.2 Assessment of biological characteristics of phage P2-71

**Figure 2:**
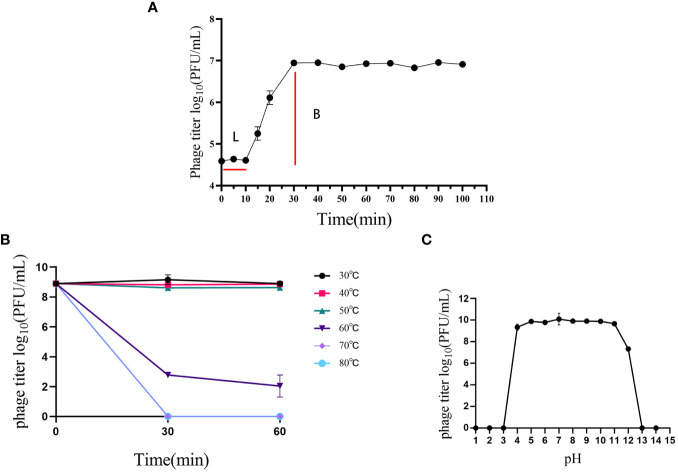
Biological characterization of phage P2-71. **(A)** Growth Kinetics: The one-step growth curve of phage P2-71 showing an initial latency phase of 10 minutes followed by a rapid rise phase over the next 20 minutes, leading to a plateau that persists for about 70 minutes, indicative of the phage’s replication cycle within the host bacteria. **(B)** Thermal Stability: Phage P2-71 demonstrates consistent phage titers indicating stability from 30°C to 50°C. At 60°C, a marked decline in phage titer suggests a limit to thermal tolerance, beyond which phage activity is compromised. **(C)** pH Stability: Stability testing across a pH gradient shows that phage P2-71 maintains stability within a pH range of 4 to 11, with a slight reduction in activity observed at pH 12, demonstrating the phage’s adaptability to various pH conditions. Error bars represent the standard deviation (SD) from three independent experiments.

The one-step growth curve of phage P2-71 was characterized by a 10-minute latency phase, succeeded by a 20-minute exponential rise phase, culminating in a plateau phase that was sustained for approximately 70 minutes. The estimated burst size was determined to be 228 phage particles per infected bacterium (**Figure 2A**).

Thermal stability assays revealed that phage P2-71 maintained stable titers between 30°C and 50°C. A discernible decrease in activity was observed at 60°C, indicating a threshold for thermal stability (**Figure 2B**).

Furthermore, the phage’s resilience across a broad pH spectrum was evaluated. Phage P2-71 displayed considerable stability within a pH range of 4 to 11, with marginal activity persisting at pH 12. This robustness to pH variations underscores the potential for versatile applications of phage P2-71 (**Figure 2C**).

### 3.3 Killing assay

**Figure 3:**
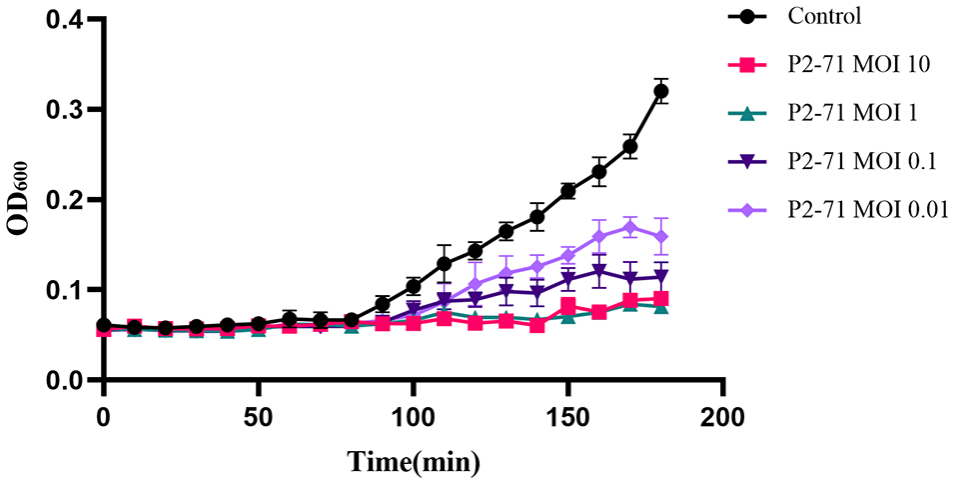
Killing assay of phage P2-71 to lyse *P. mirabilis* 37. This figure presents the effect of phage P2-71 on P. mirabilis 37 growth at varying multiplicities of infection (MOIs) of 10, 1, 0.1, and 0.01. The control group (black line) consists of bacterial culture without phage treatment. Each line represents the change in optical density at 600 nm (OD600) over time, indicating bacterial growth. A noticeable reduction in bacterial proliferation is evident in cultures treated with phage P2-71, with the degree of inhibition correlating to the MOI used. The lowest MOI of 0.01 still exhibits noticeable suppression of bacterial growth. Data points are the mean of triplicate measurements, with error bars indicating SD. These results illustrate the potent bactericidal activity of phage P2-71 across a range of MOIs.

Logarithmic phase cultures of *P. mirabilis* 37 were treated with phage P2-71 at MOIs of 10, 1, 0.1, and 0.01. Optical density at 600 nm (OD600) was periodically measured using a spectral scanning multimode reader to monitor bacterial growth. The resulting killing curves demonstrated a progressive inhibition of bacterial proliferation post-phage addition, with the extent of reduction being directly proportional to the respective MOI. Remarkable bacterial suppression was observed consistently, even at the lowest tested MOI of 0.01 (**Figure 3**).

### 3.4 Host range

Different MDR strains of *P. mirabilis* and four standard strains of bacteria (*Salmonella enterica* H9812, *Pseudomonas aeruginosa* PAO1, *Staphylococcus aureus* ATCC, 25923, and *enterotoxigenic Escherichia coli* ATCC, 25922 (ETEC) were assessed using double layer agar plate method. The results revealed that phage P2-71 has lysis ability against a portion of MDR strains of *P. mirabilis* (9/36) but does not exhibit the lysis ability to the four Standard strains of bacteria mentioned above (**Supplementary Table 3**).

### 3.5 Stability of phage P2-71 in artificial urine

**Figure 4:**
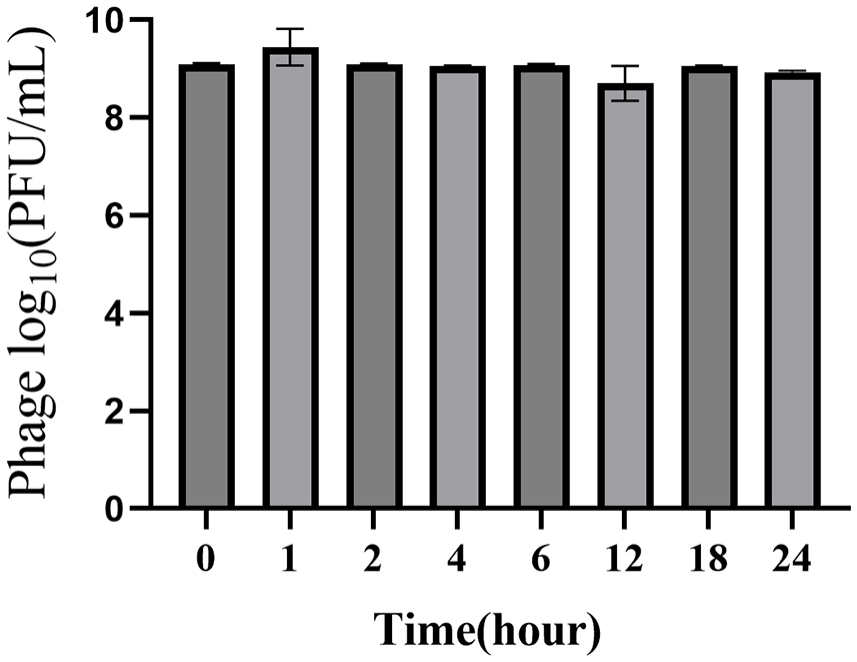
Stability of phage P2-71 over a 24-hour period in artificial urine. The bar chart illustrates the titer of bacteriophage P2-71 at various time points following inoculation into artificial urine. Samples were collected immediately after inoculation (0 hours) and subsequently at 1, 2, 4, 6, 12, 18, and 24 hours post-inoculation. Error bars represent the SD from three independent replicates. Statistical analysis using one-way ANOVA revealed that variations in phage titers over the 24-hour period were not statistically significant, indicating consistent stability of phage P2-71 in artificial urine.

The stability assessment of phage P2-71 over a 24-hour period in artificial urine demonstrated no significant change in phage concentration. The titer remained stable across all measured time points, including 1, 2, 4, 6, 12, 18, and 24 hours post-inoculation. Statistical analysis using one-way ANOVA confirmed that the observed variations in the data were within the standard deviation range, and the differences between groups did not reach statistical significance, with p-values greater than 0.05 (**Figure 4**).

### 3.6 Time-kill assay of P2-71 in artificial urine

**Figure 5:**
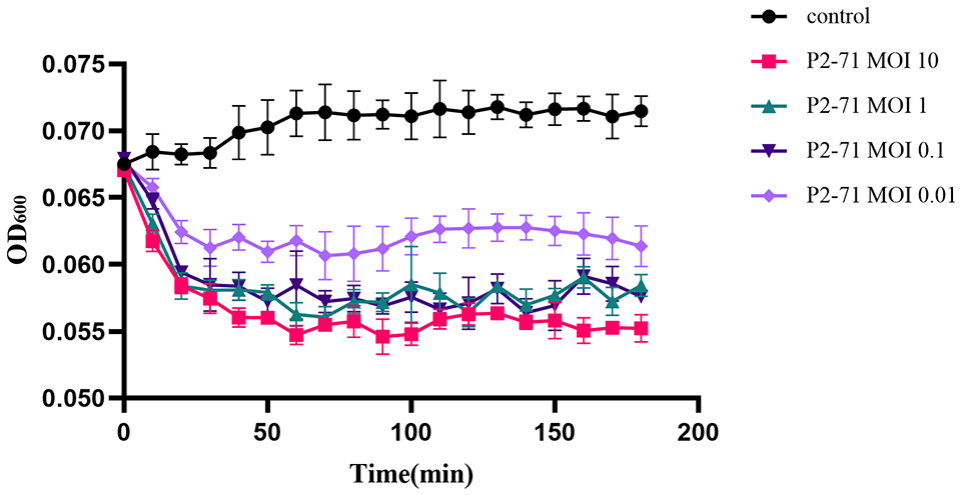
Killing assay of phage P2-71 to lyse *P. mirabilis* 37 in artificial urine. This graph displays the OD600 measured over 180 minutes following inoculation. The control group (black) shows a slight uptrend in OD600, indicative of minimal bacterial growth. In contrast, the phage-treated groups experience an immediate decline in OD600, with the highest MOI of 10 (red) showing a marked reduction in bacterial growth. Following this initial drop, the OD600 values for the phage-treated samples plateau, indicating a state of equilibrium between phage activity and bacterial growth. Error bars denote SD from three replicates.

The application of phage P2-71 to *P. mirabilis* 37 cultures in artificial urine exhibited a MOI-dependent inhibition of bacterial growth. The control group showed a slight increase in OD600. In contrast, phage-treated groups demonstrated an immediate decline in OD600 after phage addition, with a more pronounced effect at the highest MOI. Following the initial reduction, OD600 values across phage-treated samples plateaued, suggesting an equilibrium between phage action and bacterial growth. Lower MOI treatments resulted in a less marked decrease in OD600 (**Figure 5**).

### 3.7 Genomic insights and comparative analysis of phage P2-71

**Figure 6:**
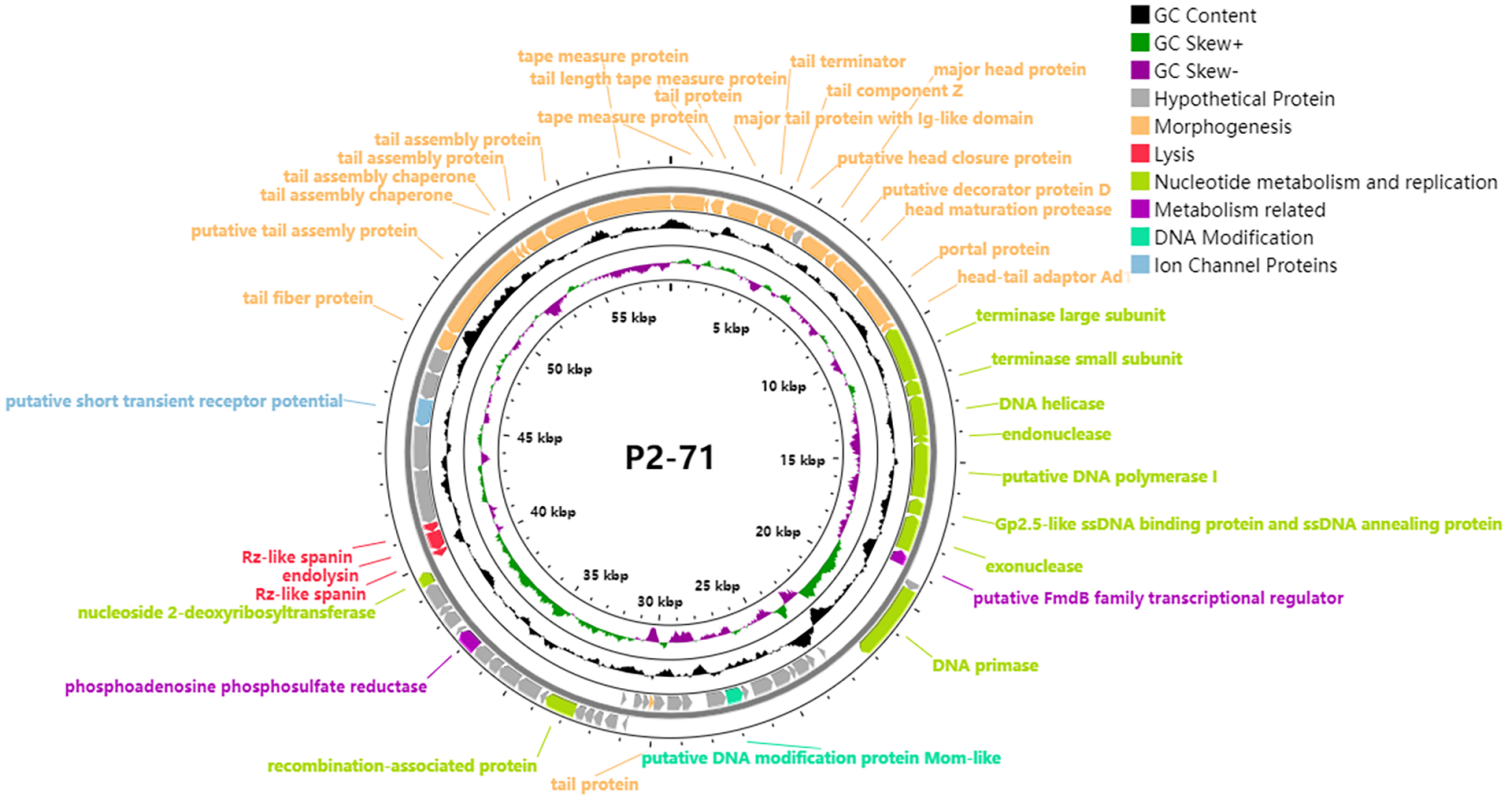
Circular genomic map of phage P2-71. This circular representation delineates the organization and functional categorization of the 71 open reading frames (ORFs) identified within the 58,706 bp genome of phage P2-71. Key functional groups are color-coded: morphogenesis proteins critical for phage structure are highlighted in yellow; lysis proteins involved in host cell disruption are marked in red; proteins associated with nucleotide metabolism and replication are in green, indicating their role in phage DNA synthesis; metabolism-related proteins are in purple, reflecting their involvement in various biochemical pathways; DNA modification proteins, potentially implicated in host immune evasion, are in cyan; ion channel proteins that may affect cellular homeostasis are in blue; and proteins of unknown function are denoted in gray. The genomic map was constructed using Prokee, providing a visual synopsis of the genetic architecture of phage P2-71. The displayed organization aids in the elucidation of the phage’s potential interactions with host cells and its overall biological functionality.

The genomic analysis of phage P2-71 has revealed a genome comprising 58,706 base pairs (bp) with a G+C content of 46.87%. Analysis of this genome has identified 71 open-reading frames (ORFs) (**Supplementary Table 4**), each classified according to their predicted functions derived from sequence analysis, enhancing our understanding of the phage’s genetic blueprint. These ORFs span a diverse array of roles, from contributing to the phage’s structural integrity to mediating critical enzymatic functions within its life cycle. Notably, ORF1 is characterized as encoding a protein that correlates with the dimensional attributes of the phage tail, an attribute likely significant in host cell recognition. ORF4 and ORF65, implicated in tail morphology, suggest a contributory role to the phage’s affinity for specific host interactions. The identification of ORF57 and ORF58 as potential encoders of lysis-associated proteins posits their involvement in the liberation of nascent phage particles following host cell infection. Additionally, the characterization of ORF31, bearing similarity to DNA-modifying enzymes, implies a possible function in the phage’s interaction with host genomic material, which may influence its replication efficacy and host immune evasion strategies. The complete genomic sequence of phage P2-71 has been deposited in the NCBI database, with the accession number OR672055.1. The genomic map (**Figure 6**) categorizes the identified proteins by their predicted functions and provides a visual overview of the phage P2-71 genome.

**Figure 7:**
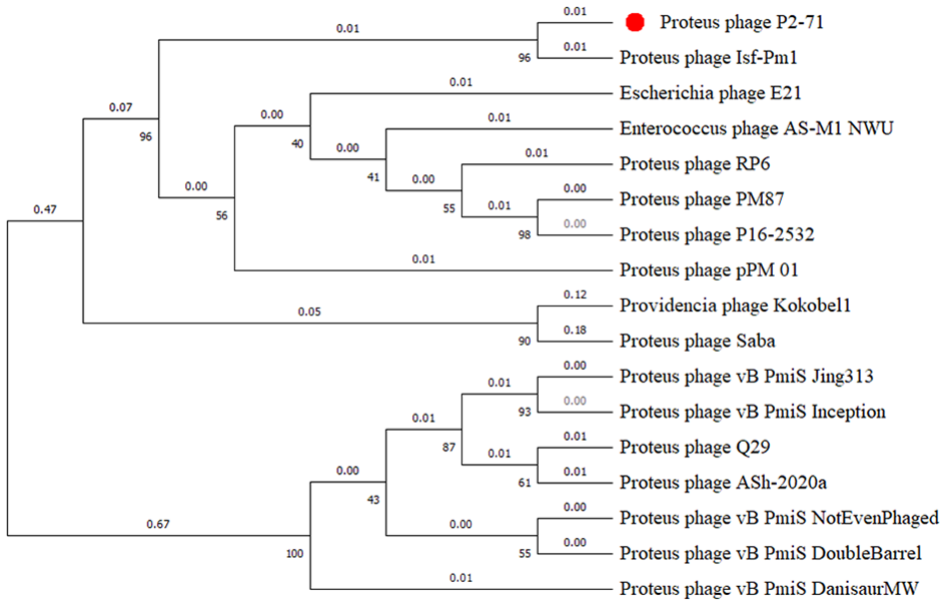
Phylogenetic tree illustrating the relationships of phage P2-71 with related phages, based on terminase large subunit gene sequences. The numbers on the branches represent the number of nucleotide substitutions per site. Bootstrap values (from, 1000 replicates) are indicated at each node, expressed as integers corresponding to percentages, to demonstrate the statistical support for each clade. Phage P2-71 is distinctly marked with a red dot for emphasis.

Our phylogenetic analysis, based on the terminase large subunit gene sequences, positions phage P2-71 within a distinct clade, denoting its close genetic relatedness with particular *Proteus* phages. The constructed evolutionary tree demonstrates P2-71 clustering closely with Proteus phage Isf-Pm1(Mirzaei et al., 2022). This clustering is supported by robust bootstrap values, which suggest a high level of confidence in this particular branch of the tree. The observed phylogenetic branching patterns and genetic distance metrics provide insights into P2-71’s evolutionary lineage and its relationships with other closely related phages (**Figure 7**).

**Figure 8:**
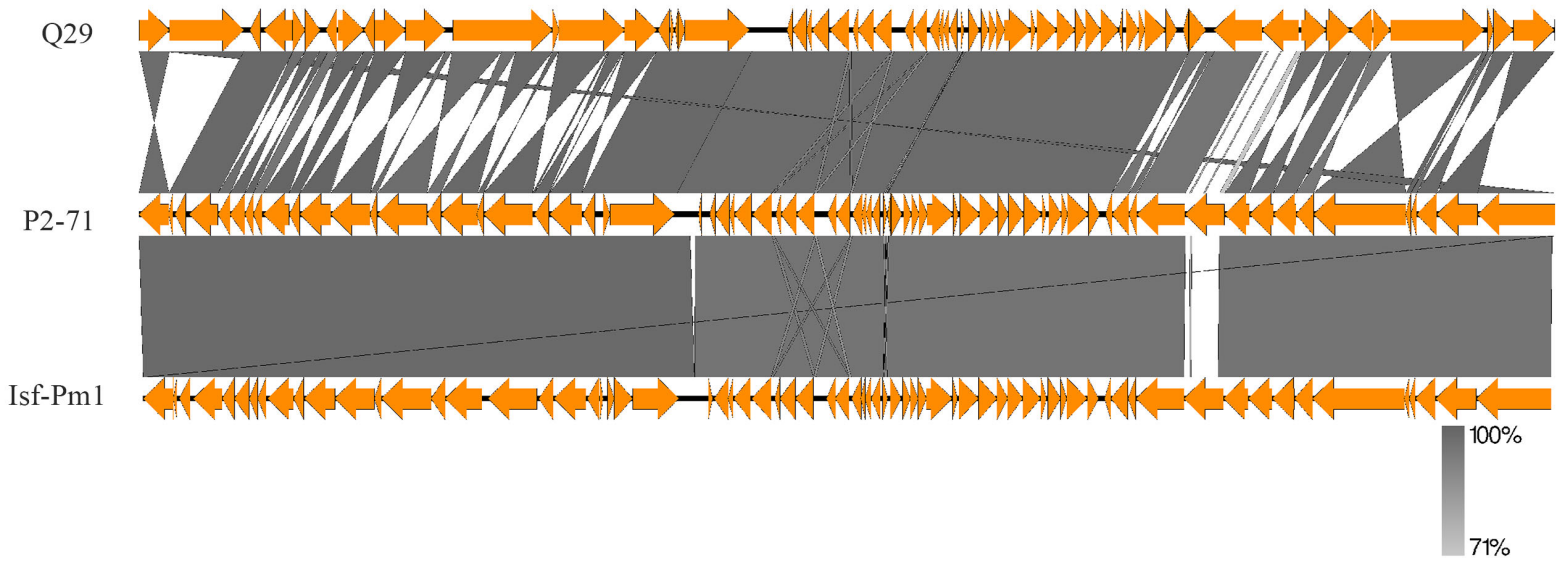
Genomic comparison of phage P2-71 with phage Q29 and phage Isf-Pm1 using Easyfig 2.2.5. The synteny plot in the upper panel showcases the genomic relationship between P2-71 and the more distantly related Q29, illustrating significant genomic rearrangement. The lower panel depicts the synteny between P2-71 and Isf-Pm1, highlighting areas of high sequence conservation. The regions of sequence identity are shaded in gray, and gene annotations are represented by arrows, which denote the direction of transcription.

Genomic comparison studies facilitated by Easyfig 2.2.5 have revealed key aspects of phage P2-71’s genetic structure. Synteny analysis between P2-71 and the closely related *Proteus* phage Isf-Pm1 demonstrates a high degree of genomic conservation, with extensive synteny blocks indicative of recent evolutionary divergence. In contrast, the comparison with the more distantly related phage Q29 shows significant genomic rearrangement and a marked reduction in conserved sequences, suggesting an evolutionary pathway with considerable divergence. These comparative genomic insights reveal the variability among phages and underscore distinctive genomic attributes of P2-71 that may influence its phage life cycle and host specificity (**Figure 8**).

## 4 Discussion

The rise of MDR bacterial strains presents a substantial risk to public health, particularly in the case of UTIs caused by MDR bacteria in elderly patients, which add a substantial burden to care (Garcia-Bustos et al., 2021). In the veterinary field, *P. mirabilis*, a pathogen associated with UTIs in companion animals, has been identified as a cause of health issues in these animals (Jacobsen et al., 2008). Significantly, there is a notable clonal relatedness in urinary pathogenic *P. mirabilis* observed across humans and their domestic pets, suggesting the potential for cross-species transfer of UTI-inducing *P. mirabilis* (Marques et al., 2019). MDR *P. mirabilis* strains have been identified in diverse animal species, including ducks (Algammal et al., 2021), cattle (Jayol et al., 2018), and chickens (Li et al., 2022). In this study, both the MDR *P. mirabilis* strain and phage p2-71 were isolated from a stray dog center. Given the close contact between stray dogs and humans, it is reasonable to speculate that MDR *P. mirabilis* could potentially be transmitted from stray dogs to humans, representing a possible risk to human health. Although our study did not detect whether individuals who came into contact with stray dogs were carriers of *P. mirabilis*, the potential risk of transmission from animals to humans cannot be overlooked. Stray dogs, as animals in close contact with humans, may act as vectors for MDR bacteria, especially in the absence of adequate hygiene measures and surveillance. This finding is particularly relevant in the field of public health, as it underscores the need for monitoring and controlling the transmission of MDR bacteria between animals and humans.

The discovery of antibiotics was a significant breakthrough in medical history, saving countless lives. However, the rise and dissemination of strains resistant to antibiotics have severely challenged the efficacy of antibiotics (Chinemerem Nwobodo et al., 2022). Resistant bacteria proliferate not only in hospital settings but are also widespread in aquatic and agricultural environments (Ondon et al., 2021; Papajová et al., 2022). These bacteria disseminate their resistance genes via Horizontal Gene Transfer (HGT), exacerbating the global issue of antibiotic resistance (Sun et al., 2019)。Consequently, the search for alternative strategies to antibiotics has become both urgent and necessary. Phage therapy, as an emerging antimicrobial strategy, has shown immense potential. Phages are highly specific, targeting only particular bacterial hosts, thereby minimizing disruption to the beneficial microbiota in the human body (Lorenzo-Rebenaque et al., 2023), and reducing the development of antibiotic resistance. In this study, we isolated phage P2-71 from the feces of stray dogs, which targets the canine-sourced MDR *P. mirabilis*. This discovery not only provides a new perspective for treating MDR bacterial infections but also lays the groundwork for further research into the application of phages in public health.

Phages infect and lyse their host bacteria through the specific recognition of receptors on the cell wall (Dunne et al., 2021). *P. mirabilis* phage Q29 has demonstrated the capability to lyse 12 out of 27 MDR *P. mirabilis* strains (Hao et al., 2023). In this study, to ascertain the host spectrum of phage P2-71, the double-layer agar technique was utilized, which was able to lyse 9 out of 36 MDR *P. mirabilis* strains preserved in our laboratory. This finding highlights its potential for application. However, it is noteworthy that our host range analysis was limited to a relatively small number of canine-sourced MDR *P. mirabilis* strains available in our laboratory collection. Therefore, the sample size was limited, and it is generally understood that a broader host range often implies a wider scope of potential applications.

The genome size of phage P2-71 is approximately 58,706 bp, containing about 71 predicted ORFs, with a GC content of 46.87%. Phage P2-71 consists of a dodecahedral head approximately 62 ± 3.0 nm in diameter, coupled with a tail measuring about 234 ± 10 nm in length. According to the ICTV classification guidelines, P2-71 belongs to the Siphoviridae family. The tape measure protein (TMP), encoded by ORF1, is a pivotal factor in determining the length of the phage tail. This length is crucial for bridging the distance between the phage and the host cell surface, thereby influencing the phage’s ability to penetrate bacterial cell envelopes and reach specific receptors. Studies on the T5 tail tube structure of Siphoviridae phages have revealed the multifunctional nature of TMP, indicating its involvement in host recognition, cell wall perforation, and viral DNA transfer to the host cytoplasm (Arnaud et al., 2017). Furthermore, the evolution of TMPs, characterized by variations in tail length due to gene duplications and losses, underscores the diversity in phage tail architectures and strategies for cell wall recognition and perforation across different phage families (Belcaid et al., 2011). This diversity potentially dictates the range of bacterial cells a phage can infect, with tail length variations influencing the phage’s infective capabilities. The presence of ORF4 and ORF65, which encode for the major tail protein with an Ig-like domain and tail fiber proteins respectively, indicates specialized mechanisms for host cell receptor recognition and binding. The Ig-like domain in the major tail protein may play a crucial role in mediating specific interactions with bacterial cell surface proteins, thereby determining the host range of P2-71 (Fraser et al., 2007; Chatterjee et al., 2021). Similarly, tail fiber proteins are instrumental in recognizing and binding to specific receptors on the bacterial surface, a vital process for successful phage attachment and subsequent infection (Nobrega et al., 2018; Farquharson et al., 2021; Taslem Mourosi et al., 2022). ORF57 and ORF58 encode key lysis proteins crucial in the final stage of the phage P2-71 infection cycle, facilitating the release of new virions. ORF58 encodes an endolysin, a phage-encoded peptidoglycan hydrolase, which first degrades the peptidoglycan layer of the bacterial cell wall from within, critical for efficient lysis in G- bacteria (Schmelcher et al., 2012; Rahman et al., 2021). Following this degradation, the Rz-like spanin encoded by ORF57 disrupts the outer membrane, a distinctive feature of G- bacterial cells, allowing the release of progeny phages (Krupovič et al., 2008; Kongari et al., 2018). The sequential action of endolysin and Rz-like spanin is essential for effectively breaching the host cell wall and facilitating the release and spread of progeny phages. Understanding the roles of ORF57 and ORF58 in this process provides valuable insights into how P2-71 adapts its infection mechanism for G- bacterial hosts, highlighting its host specificity and infection efficiency. The remaining 37 ORFs are classified as hypothetical proteins, indicating that their functions are still unclear. The unknown functions of these hypothetical proteins provide a wide scope for future research, which may reveal new mechanisms of phage-host interactions.

Phages utilized in therapeutic applications should ideally be devoid of virulence factors and antibiotic resistance genes. Such phages are more favorable for clinical use, as they minimize potential risks to the host and do not facilitate the emergence of drug resistance. In our study, phage P2-71 demonstrated an absence of detectable antibiotic resistance and virulence genes, positioning it as a viable and promising candidate for therapeutic deployment. This characteristic is particularly pertinent in the face of the escalating crisis of antibiotic resistance, underscoring the importance of phages free from resistance genes as they do not contribute to the aggravation of this global health challenge.

In our study, a phylogenetic analysis of the terminase large subunit enzyme revealed that phage P2-71 shares a close genetic relationship with specific *Proteus* phages, particularly Isf-Pm1(Mirzaei et al., 2022). This connection is supported by high bootstrap confidence values, indicating the robustness of this phylogenetic branch. The high degree of genomic conservation between P2-71 and Isf-Pm1 suggests a recent divergence event from a common ancestor. Such shared characteristics might be a consequence of gene transfer events occurring between different hosts, significantly impacting the adaptability and evolutionary trajectory of these phages. This conservation may elucidate common biological traits shared between the two, such as host range, infection mechanisms, and environmental adaptability. However, the growth curve of Isf-Pm1, with a latency phase lasting 60 minutes and a burst yield of 46 phages per bacterium, differ markedly from those of P2-71, which demonstrates an unusually brief latent period of merely 10 minutes and a notably high burst size, producing 228 phage particles for each infected cell. In terms of growth curves, significant differences were observed between the two phages. Regrettably, the stability and other biological characteristics of Isf-Pm1 have not yet been documented in the literature. Furthermore, a genomic comparison of P2-71 with another phage, Q29 (Hao et al., 2023), revealed substantial rearrangements and divergences, hinting at different evolutionary strategies adopted by phages to adapt to host defense mechanisms and environmental pressures (Dion et al., 2020; Zhang et al., 2023). This genomic plasticity may indicate phages’ ability to adapt to variable environments. The marked differences from Q29 might also reflect unique mechanisms employed by P2-71 in its interactions with hosts. The genomic analysis of P2-71 reveals characteristics that might suggest a broader host range potential. However, this must be interpreted in the context of our empirical data, which showed that P2-71 lysed 25% (9 out of 36) of the tested *P. mirabilis* isolates. While these genomic insights hint at wider applications in phage therapy, particularly amid escalating antibiotic resistance, they also underline the necessity for more exhaustive research to fully understand P2-71’s infection strategies and precise host range. Our study’s exploration into the genetic diversity and evolutionary dynamics of P2-71 provides vital insights, laying a foundation for future research aimed at deciphering how these factors influence the efficacy and specificity of phage therapy against various bacterial strains.

Phage P2-71 demonstrated exceptional thermal stability, maintaining stability across a temperature range of 30°C to 50°C and retaining partial activity even at temperatures as high as 60°C. Such thermal stability is particularly crucial in phage therapy applications, especially considering the potential impact of temperature fluctuations within the human body on phage efficacy. In contrast, phage Q29 (Hao et al., 2023) maintained partial activity at 65°C, while vB_PmiS-TH (Yazdi et al., 2018) exhibited certain activity even at 75°C, indicating a broader range of temperature adaptability. Regarding pH stability, P2-71 remained relatively stable from pH 4 to 11 and showed some activity even at pH 12. This wide pH stability implies that P2-71 can effectively operate under diverse environmental conditions, which is vital for therapeutic applications due to the significant pH variations in different parts of the human body. By comparison, Q29 maintained a high titer within the pH range of 4 to 11, whereas vB_PmiS-TH sustained activity from pH 3 to 11 but exhibited a significant decline in activity after 24 hours. The latent period of P2-71 is only 10 minutes, with its proliferation rate rapidly escalating within 20 minutes and then maintaining a stable growth phase for approximately 70 minutes. Its burst size is 228 phage particles released per infected cell. This rapid replication capability and substantial burst size mean that P2-71 can quickly proliferate and release a large number of new phages upon infecting host cells, crucial for the swift eradication of pathogens. In comparison, the burst sizes for Isf-Pm1 and Isf-Pm (Mirzaei et al., 2022) are 46 and 666 particles, respectively, while vB_PmiS-TH shows a burst size of 260 PFU per infected cell, similar to P2-71, demonstrating the variability in replication capacities of different phages within host cells. Overall, the biological characteristics of phage P2-71, including its excellent thermal stability, wide pH stability, and large burst size, make it a highly promising candidate for therapeutic applications.

The observed stability of phage P2-71 in artificial urine over 24 hours underscores its potential for phage therapy applications in the urinary tract. This stability, without significant titer fluctuations, aligns with Pereira et al (Pereira et al., 2016), who found phages maintain their concentration in urine, even increasing in the presence of host bacteria, though with diminished inactivation efficacy compared to PBS. In LB medium, the phage P2-71 showcases a potent inhibitory effect on bacterial growth that is maintained throughout the duration of the experiment. The highest MOI of 10 results in a significant and sustained reduction in OD600, indicating a strong and persistent bactericidal activity. This effect is consistent across the various MOIs tested, with even the lowest MOI of 0.01 showing a considerable suppression of bacterial proliferation. Conversely, in the artificial urine environment, the initial impact of the phage is more abrupt, with a rapid decrease in OD600 noted immediately following phage addition. This suggests a swift onset of phage activity. However, unlike in LB medium, the magnitude of bacterial growth suppression is less intense, and a plateau is reached relatively quickly. This plateau phase could suggest a dynamic equilibrium where the bactericidal action of the phage might be counterbalanced by bacterial regrowth or the potential onset of resistance mechanisms within the bacterial population. The differential responses observed could be attributed to the nutrient composition of the two media. The rich nutrient environment of LB medium may potentially support more vigorous phage replication and subsequent bacterial lysis. The phage-bacteria interactions observed in artificial urine may mirror the intricate dynamics within the urinary tract, akin to the comprehensive overview provided by Żaczek et al (Żaczek et al., 2020), highlighting the significance of comprehending phage-host interplay across different bodily fluids. Additionally, it is crucial to consider these findings in the context of studies like Zulk et al (Zulk et al., 2022), which reveal the complexities of phage behavior in more natural environments such as human urine. Zulk et al. observed a reduction in phage lytic activity and the rapid emergence of phage-resistant bacterial strains in human urine. However, interestingly, these phage-resistant bacteria might be more susceptible to certain antibiotics, providing a theoretical basis for the synergistic use of phages and antibiotics, which could have significant clinical implications.​The stability of P2-71 in artificial urine suggests its potential for long-term treatment of UTIs. This stability implies that P2-71 could remain effective in the complex environment of urine, providing a sustained therapeutic approach. This is particularly significant for UTI cases requiring long-term management, as the stable activity of the phage helps maintain consistent therapeutic effects over time. While our study demonstrates the stability of P2-71 in artificial urine, a notable limitation is the absence of investigation into bacterial resistance development against this phage. As underscored by Oromí-Bosch et al (Oromí-Bosch et al., 2023), understanding and addressing bacterial resistance is crucial for the effective application of phage therapy​​. The potential for bacteria to develop resistance over time to P2-71, and the strategies to mitigate such resistance, remain unexplored in our research. Future studies are therefore essential to examine the resistance dynamics specific to P2-71 and to develop methods that could sustain its efficacy, such as phage cocktails, phage engineering, and phage-antibiotic synergy. Addressing these aspects is critical to ascertain the long-term clinical applicability of P2-71 in UTI treatment.

In summary, our study successfully isolated the lytic *P. mirabilis* phage P2-71 and provided a detailed description of its biological properties and genome. These findings not only offer valuable insights into phage therapy as a potential alternative to antibiotic treatments but also hold significant implications for the management of UTIs caused by MDR bacteria in human and veterinary healthcare.

## Data availability statement

The data presented in the study are deposited in the NCBI repository, accession number OR672055.

## Ethics statement

The animal study was approved by The Sichuan Agricultural University’s Institutional Animal Care and Use Committee. The study was conducted in accordance with the local legislation and institutional requirements.

## Author contributions

ZD: Formal analysis, Investigation, Methodology, Writing – original draft. RW: Investigation, Writing – original draft. LL: Software, Writing – original draft. SA: Software, Writing – original draft. JY: Data curation, Writing – original draft. QL: Funding acquisition, Resources, Supervision, Writing – original draft. KF: Funding acquisition, Resources, Supervision, Writing – original draft. YZ: Funding acquisition, Resources, Supervision, Writing – original draft. HF: Project administration, Validation, Writing – review & editing. ZYZ: Project administration, Validation, Writing – review & editing. HL: Project administration, Validation, Writing – review & editing. ZJZ: Project administration, Validation, Writing – review & editing. XQ: Conceptualization, Writing – review & editing. GP: Conceptualization, Methodology, Writing – review & editing.
