# Supplementary material for: Phage P2-71 against multi-drug resistant Proteus mirabilis: isolation, characterization, and non-antibiotic antimicrobial potential
Source: Front Cell Infect Microbiol. 2024 Mar 4;14:1347173. doi: 10.3389/fcimb.2024.1347173 (PMC10945010; doi:10.3389/fcimb.2024.1347173)
Supplement: Supplementary file 1 [file Table_1.docx]

**Supplementary material**

# Supplementary Table 1. An overview of all the bacteria used for the host range of phage P2-71

| Strain | Source | Accession Number | Antimicrobial resistance phenotypes |
| --- | --- | --- | --- |
| *Proteus mirabilis* 4 | Dog feces | OP393034 | AMP/AMC/SAM/KZ/FEP/CTX/IPM/MEM/CN/TE/SXT/FOS/C |
| *Proteus mirabilis* 7 | Dog feces | OP393037 | AMP/AMC/KZ/FEP/CTX/MEM/TE/SXT/C |
| *Proteus mirabilis* 8 | Dog feces | OP393038 | AMP/AMC/SAM/KZ/FEP/CTX/TE/CIP/SXT/FOS/C |
| *Proteus mirabilis* 9 | Dog feces | OP393039 | AMP/AMC/SAM/KZ/FEP/CTX/IPM/CN/TE/CIP/SXT/C |
| *Proteus mirabilis* 10 | Dog feces | OP393040 | IPM/TE/SXT/C |
| *Proteus mirabilis* 15 | Dog feces | OP393045 | AMP/AMC/SAM/KZ/FEP/CTX/IPM/TE/CIP/SXT/C |
| *Proteus mirabilis* 17 | Dog feces | OP393047 | KZ/IPM/TE/CIP/SXT/C |
| *Proteus mirabilis* 18 | Dog feces | OP393048 | KZ/IPM/TE/CIP/SXT/C |
| *Proteus mirabilis* 19 | Dog feces | OP393049 | AMP/AMC/KZ/FEP/CTX/TE/SXT/FOS |
| *Proteus mirabilis* 20 | Dog feces | OP393050 | AMP/AMC/SAM/KZ/IPM/CN/TE/CIP/SXT/C |
| *Proteus mirabilis* 21 | Dog feces | OP393072 | AMP/AMC/SAM/KZ/FEP/CTX/CN/TE/CIP/SXT/FOS/C |
| *Proteus mirabilis* 22 | Dog feces | OP393073 | AMP/KZ/CTX/TE/CIP/SXT/F/FOS/C |
| *Proteus mirabilis* 23 | Dog feces | OP393074 | AMP/SAM/KZ/FEP/CTX/IPM/MEM/CN/TE/CIP/SXT/FOS/C |
| *Proteus mirabilis* 24 | Dog feces | OP393075 | AMP/KZ/TE/SXT/FOS |
| *Proteus mirabilis* 25 | Dog feces | OP393076 | AMP/AMC/SAM/KZ/FEP/CTX/IPM/MEM/CN/TE/CIP/SXT/F/FOS/C |
| *Proteus mirabilis* 26 | Dog feces | OP393077 | AMP/SAM/KZ/FEP/CTX/ATM/CN/TE/CIP/SXT/FOS/C |
| *Proteus mirabilis* 27 | Dog feces | OP393078 | AMP/SAM/KZ/IPM/TE/F |
| *Proteus mirabilis* 28 | Dog feces | OP393079 | AMP/KZ/IPM/TE |
| *Proteus mirabilis* 29 | Dog feces | OP393080 | AMP/KZ/TE/F/FOS |
| *Proteus mirabilis* 30 | Dog feces | OP393081 | AMP/SAM/KZ/IPM/MEM/TE/CIP/SXT/C |
| *Proteus mirabilis* 31 | Dog feces | OP393082 | AMP/KZ/TE/C |
| *Proteus mirabilis* 32 | Dog feces | OP393083 | AMP/AMC/KZ/IPM/TE/F/C |
| *Proteus mirabilis* 34 | Dog feces | OP393085 | AMP/KZ/CTX/CN/TE/CIP/SXT/FOS/C |
| *Proteus mirabilis* 35 | Dog feces | OP393086 | AMP/KZ/CTX/TE/CIP/SXT/FOS/C |
| *Proteus mirabilis* 36 | Dog feces | OP393087 | IPM/TE/SXT/F/C |
| *Proteus mirabilis* 37 | Dog feces | OP393088 | KZ/IPM/MEM/TE/F |
| *Proteus mirabilis* 38 | Dog feces | OP393089 | KZ/MEM/TE/FOS |
| *Proteus mirabilis* 43 | Dog feces | OP393094 | AMP/KZ/IPM/TE/FOS |
| *Proteus mirabilis* 45 | Dog feces | OP393096 | AMP/KZ/IPM/TE/SXT |
| *Proteus mirabilis* 58 | Dog feces | OP393054 | KZ/IPM/TE/FOS |
| *Proteus mirabilis* 63 | Dog feces | OP393059 | KZ/IPM/TE/FOS |
| *Proteus mirabilis* 69 | Dog feces | OP393065 | KZ/FEP/IPM/TE/FOS |
| *Proteus mirabilis* 70 | Dog feces | OP393066 | FEP/IPM/TE/FOS |
| *Proteus mirabilis* 71 | Dog feces | OP393067 | KZ/MEM/TE/FOS |
| *Proteus mirabilis* 72 | Dog feces | OP393068 | KZ/IPM/MEM/TE/FOS |
| *Proteus mirabilis* 73 | Dog feces | OP393069 | KZ/IPM/TE/FOS |
| *Proteus mirabilis* 75 | Dog feces | OP393071 | KZ/IPM/TE/FOS |
| *Salmonella enterica* H9812 | Standard strain |  |  |
| *Pseudomonas aeruginosa* PAO1 | Standard strain |  |  |
| *Staphylococcus aureus* ATCC 25923 | Standard strain |  |  |
| *enterotoxigenic Escherichia* coli ATCC 25922 | Standard strain |  |  |

The table provides a detailed overview of all bacterial strains utilized for host range testing of phage P2-71. Listed are the strain designations, their source of isolation, and the GenBank accession numbers corresponding to the amplified 16S rRNA gene sequences. Additionally, the table provides the antimicrobial resistance phenotypes of each strain, which indicate their resistance profile against a range of antibiotics, such as Ampicillin (AMP), Amoxicillin/Clavulanic Acid (AMC), and others. The detailed resistance patterns outlined here are critical for understanding the breadth of phage P2-71's efficacy against multi-drug resistant bacteria.

# Supplementary Table 2. Phages used for genomic analysis and phylogenetic tree construction

| Name | Size (bp) | Accession number |
| --- | --- | --- |
| Phage P2-71 | 58706 bp | OR672055.1 |
| *Proteus* phage Isf-Pm1 | 58354 bp | OL741431.1 |
| *Escherichia* phage E21 | 58536 bp | NC_052983.1 |
| *Enterococcus* phage AS-M1 NWU | 58683 bp | OR506460.1 |
| *Proteus* phage RP6 | 58619 bp | ON529532.1 |
| *Proteus* phage PM87 | 59128 bp | NC_052981.1 |
| *Proteus* phage P16-2532 | 58931 bp | MN840486.1 |
| *Proteus* phage pPM_01 | 58546 bp | NC_028812.1 |
| *Providencia* phage Kokobel1 | 59837 bp | NC_052979.1 |
| *Proteus* phage Saba | 60056 bp | NC_052978.1 |
| *Proteus* phage vB_PmiS_Jing313 | 58534 bp | OK499975.1 |
| *Proteus* phage vB_PmiS_Inception | 58589 bp | OK499974.1 |
| *Proteus* phage Q29 | 58663 bp | OM962992.1 |
| *Proteus* phage Ash-2020a | 59078 bp | NC_052980.1 |
| *Proteus* phage vB_PmiS_NotEvenPhaged | 58566 bp | OK499986.1 |
| *Proteus* phage vB_PmiS_DoubleBarrel | 59089 bp | OK500000.1 |
| *Proteus* phage vB_PmiS_DanisaurMW | 58538 bp | OK499998.1 |

This table lists the phages compared in the study, including their genome sizes and corresponding GenBank accession numbers. These sequences were utilized to ascertain the phylogenetic position of phage P2-71 relative to other bacteriophages.

# Supplementary Table 3. Host range of phage P2-71

| Strain | Lytic | Strain | Lytic |
| --- | --- | --- | --- |
| *Proteus mirabilis* 1 | - | *Proteus mirabilis* 22 | + |
| *Proteus mirabilis* 4 | - | *Proteus mirabilis* 23 | - |
| *Proteus mirabilis* 7 | + | *Proteus mirabilis* 24 | - |
| *Proteus mirabilis* 8 | - | *Proteus mirabilis* 25 | - |
| *Proteus mirabilis* 9 | - | *Proteus mirabilis* 26 | + |
| *Proteus mirabilis* 15 | - | *Proteus mirabilis* 27 | - |
| *Proteus mirabilis* 17 | - | *Proteus mirabilis* 28 | + |
| *Proteus mirabilis* 18 | + | *Proteus mirabilis* 29 | - |
| *Proteus mirabilis* 19 | - | *Proteus mirabilis* 30 | + |
| *Proteus mirabilis* 20 | - | *Proteus mirabilis* 31 | - |
| *Proteus mirabilis* 43 | + | *Proteus mirabilis* 32 | - |
| *Proteus mirabilis* 45 | - | *Proteus mirabilis* 34 | - |
| *Proteus mirabilis* 58 | - | *Proteus mirabilis* 35 | - |
| *Proteus mirabilis* 63 | + | *Proteus mirabilis* 36 | - |
| *Proteus mirabilis* 69 | - | *Proteus mirabilis* 37 | + |
| *Proteus mirabilis* 70 | - | *Proteus mirabilis* 38 | - |
| *Proteus mirabilis* 71 | - | *Salmonella enterica* H9812 | - |
| *Proteus mirabilis* 72 | - | *Pseudomonas aeruginosa* PAO1 | - |
| *Proteus mirabilis* 73 | - | *Staphylococcus aureus* ATCC 25923 | - |
| *Proteus mirabilis* 21 | - | enterotoxigenic *Escherichia coli* ATCC 25922 (ETEC) | - |

The table presents the results of the lytic activity assessment of phage P2-71 against a panel of Proteus mirabilis strains and four standard bacterial strains. Lytic activity is indicated by a positive sign (+), while a negative sign (-) denotes no lytic activity observed.

# Supplementary Table 4. Comprehensive list of open reading frames (ORFs) identified in the genome of phage P2-71

| ORFs | Start | Stop | Length(bp) | Orientation (+/-) | Predicted Function |
| --- | --- | --- | --- | --- | --- |
| ORF1 | 1 | 1254 | 1254 | - | tape measure protein |
| ORF2 | 1244 | 1402 | 159 | - | tail length tape measure protein |
| ORF3 | 1495 | 1947 | 453 | - | tail protein |
| ORF4 | 2086 | 3273 | 1188 | - | major tail protein with Ig-like domain |
| ORF5 | 3277 | 3789 | 513 | - | tail terminator |
| ORF6 | 3779 | 4396 | 618 | - | tail component Z |
| ORF7 | 4399 | 4752 | 354 | - | putative head closure protein |
| ORF8 | 4752 | 5099 | 348 | - | hypothetical protein |
| ORF9 | 5163 | 6230 | 1068 | - | major head protein |
| ORF10 | 6243 | 6659 | 417 | - | putative decorator protein D |
| ORF11 | 6673 | 7950 | 1278 | - | head maturation protease |
| ORF12 | 7947 | 9590 | 1644 | - | portal protein |
| ORF13 | 9590 | 9865 | 276 | - | head-tail adaptor Ad1 |
| ORF14 | 9878 | 11944 | 2067 | - | terminase large subunit |
| ORF15 | 11937 | 12521 | 585 | - | terminase small subunit |
| ORF16 | 12524 | 14047 | 1524 | - | DNA helicase |
| ORF17 | 14007 | 14294 | 288 | - | endonuclease |
| ORF18 | 14294 | 16327 | 2034 | - | putative DNA polymerase I |
| ORF19 | 16392 | 16997 | 606 | - | Gp2.5-like ssDNA binding protein and ssDNA annealing protein |
| ORF20 | 17029 | 18357 | 1329 | - | exonuclease |
| ORF21 | 18418 | 18900 | 483 | - | putative FmdB family transcriptional regulator |
| ORF22 | 19253 | 19537 | 285 | + | hypothetical protein |
| ORF23 | 19537 | 22212 | 2676 | + | DNA primase |
| ORF24 | 23191 | 23319 | 129 | - | hypothetical protein |
| ORF25 | 23662 | 23901 | 240 | - | hypothetical protein |
| ORF26 | 23898 | 24476 | 579 | - | hypothetical protein |
| ORF27 | 24473 | 24685 | 213 | - | hypothetical protein |
| ORF28 | 24699 | 25388 | 690 | - | hypothetical protein |
| ORF29 | 25438 | 26238 | 801 | - | hypothetical protein |
| ORF30 | 26400 | 26621 | 222 | - | hypothetical protein |
| ORF31 | 26618 | 27256 | 639 | - | putative DNA modification protein Mom-like |
| ORF32 | 27260 | 28000 | 741 | - | hypothetical protein |
| ORF33 | 28549 | 28914 | 366 | - | hypothetical protein |
| ORF34 | 28907 | 29476 | 570 | - | hypothetical protein |
| ORF35 | 29582 | 29983 | 402 | - | hypothetical protein |
| ORF36 | 29964 | 30152 | 189 | - | tail protein |
| ORF37 | 30142 | 30384 | 243 | - | hypothetical protein |
| ORF38 | 30396 | 30722 | 327 | - | hypothetical protein |
| ORF39 | 30867 | 31001 | 135 | + | hypothetical protein |
| ORF40 | 31031 | 31162 | 132 | - | hypothetical protein |
| ORF41 | 31194 | 31625 | 432 | + | hypothetical protein |
| ORF42 | 31707 | 32018 | 312 | + | hypothetical protein |
| ORF43 | 32024 | 32344 | 321 | + | hypothetical protein |
| ORF44 | 32338 | 32685 | 348 | + | hypothetical protein |
| ORF45 | 32672 | 33760 | 1089 | + | recombination-associated protein |
| ORF46 | 33763 | 33963 | 201 | + | hypothetical protein |
| ORF47 | 34030 | 34848 | 819 | + | hypothetical protein |
| ORF48 | 34845 | 35612 | 768 | + | hypothetical protein |
| ORF49 | 35609 | 36055 | 447 | + | hypothetical protein |
| ORF50 | 36048 | 36680 | 633 | + | hypothetical protein |
| ORF51 | 36680 | 37417 | 738 | + | phosphoadenosine phosphosulfate reductase |
| ORF52 | 37432 | 37596 | 165 | + | hypothetical protein |
| ORF53 | 37719 | 38252 | 534 | + | hypothetical protein |
| ORF54 | 38245 | 38496 | 252 | + | hypothetical protein |
| ORF55 | 38498 | 39361 | 864 | + | hypothetical protein |
| ORF56 | 39364 | 39828 | 465 | + | nucleoside 2-deoxyribosyltransferase |
| ORF57 | 40078 | 40332 | 255 | - | Rz-like spanin |
| ORF58 | 40332 | 41036 | 705 | - | endolysin |
| ORF59 | 41047 | 41361 | 315 | - | Rz-like spanin |
| ORF60 | 41372 | 43357 | 1986 | - | hypothetical protein |
| ORF61 | 43361 | 45001 | 1641 | - | hypothetical protein |
| ORF62 | 45011 | 46027 | 1017 | - | putative short transient receptor potential |
| ORF63 | 46040 | 47023 | 984 | - | hypothetical protein |
| ORF64 | 47034 | 47954 | 921 | - | hypothetical protein |
| ORF65 | 47954 | 48697 | 744 | - | tail fiber protein |
| ORF66 | 48697 | 52521 | 3825 | - | putative tail assemly protein |
| ORF67 | 52514 | 52723 | 210 | - | tail assembly chaperone |
| ORF68 | 52723 | 52959 | 237 | - | tail assembly chaperone |
| ORF69 | 52972 | 53787 | 816 | - | tail assembly protein |
| ORF70 | 53808 | 55496 | 1689 | - | tail assembly protein |
| ORF71 | 55502 | 58705 | 3204 | - | tape measure protein |

The table presents a comprehensive list of open reading frames (ORFs) identified in the genome of phage P2-71. For each ORF, the table provides detailed information including start and stop positions, length (in base pairs), orientation, and predicted function based on sequence homology. The functional categories assigned to these ORFs include morphogenesis, nucleotide metabolism and replication, lysis, ion channel activity, and proteins related to DNA modification. ORFs that could not be assigned to specific functional categories based on sequence homology are classified as hypothetical proteins, highlighting potential new areas for research into phage biology and function.
